# DPY19L3 promotes vasculogenic mimicry by its *C*-mannosyltransferase activity

**DOI:** 10.32604/or.2023.030304

**Published:** 2024-03-20

**Authors:** HASSAN BAYDOUN, YUJI KATO, HIROKI KAMO, ANNA HÜSCH, HAYATO MIZUTA, RYOTA KAWAHARA, SIRO SIMIZU

**Affiliations:** 1 Department of Applied Chemistry, Faculty of Science and Technology, Keio University, Yokohama, 223-8522, Japan; 2 Department of Pharmacy and Biochemistry, Faculty of Science, University of Tübingen, Tübingen, 72074, Germany

**Keywords:** *C*-mannosylation, Vasculogenic mimicry, DPY19L3

## Abstract

*C*-mannosylation is a post-translational modification that occurs intracellularly in the endoplasmic reticulum. In humans, biosynthesis of *C*-mannosylation in proteins containing thrombospondin type 1 repeat is catalyzed by the DPY19 family; nonetheless, biological functions of protein *C*-mannosylation are not yet fully understood, especially in tumor progression. Vasculogenic mimicry (VM) is the formation of fluid-conducting channels by highly invasive and genetically deregulated tumor cells, enabling the tumors to form matrix-embedded vasculogenic structures, containing plasma and blood cells to meet the metabolic demands of rapidly growing tumors. In this study, we focused on DPY19L3, a *C*-mannosyltransferase, and aimed to unravel its role in VM. Knockout of *DPY19L3* inhibited the formation of VM in HT1080 human fibrosarcoma cells. Re-expression of wild-type DPY19L3 recovered VM formation; however, DPY19L3 isoform2, an enzymatic activity-defect mutant, did not restore it, suggesting that the *C*-mannosyltransferase activity of DPY19L3 is crucial to its function. Furthermore, the knockdown of *DPY19L3* in MDA-MB-231 breast cancer cells hindered its network formation ability. Altogether, our findings suggest that DPY19L3 is required for VM formation and stipulate the relevance of *C*-mannosylation in oncogenesis.

## Introduction

*C*-mannosylation is a distinctive post-translational alteration of tryptophan residue embedded in soluble and membrane proteins [1,2]. It has been reported that *C*-mannosylation of ribonuclease 2 and *C*-mannosyl tryptophan were segregated from human urine in 1994 as the first reports of this customization [3,4]. As for the mechanism, a monomeric α-mannose is adhered to the first Trp in the Trp-x-x-Trp/Cys motif of substrate proteins by the action of *C*-mannosyltransferases in the endoplasmic reticulum [2,3]. *C*-mannosylation makes hydrophobic Trp hydrophilic; hence, the modification must affect structural conformation [5], stability [6], protein-protein interaction and enzymatic activity [5]. Around 30 proteins have been known to be *C*-mannosylated [2]. On the other hand, nematode DPY19 has been identified as *C*-mannosyltransferase for proteins containing thrombospondin type 1 repeat (TSR) [7], and its orthologs, DPY19L1 and DPY19L3, possess *C*-mannosyltransferase activity for TSR in mammalian cells [8,9], whereas, so far, there are no reports concerning DPY19L2 and DPY19L4. Although DPY19 family proteins are known to be *C*-mannosyltransferases, their roles in biological processes, such as differentiation and proliferation, are not fully understood. Because some *C*-mannosylated substrates are cancer-related proteins [5,6], it is suggested that *C*-mannosylation is associated with cancer progression.

In 1999, Maniotis and colleagues described a downright neoteric blood supply in malignant melanoma and noticed how stem-like cancer cells transdifferentiate into an endothelial-like cell phenotype [10]. Such angiogenesis-independent tumor demeanor is thought to be provided by a process known as vasculogenic mimicry (VM). VM is the genesis of microvascular channels by aggressive, metastatic, and genetically untrammeled tumor cells. In comparison to angiogenesis, the process occurs *de novo* bereft of endothelial cells; tumor cells line tumor vessels, dramatically mimicking a true vascular endothelium. Tumor cells form vessel-like complexes that are defined by a dense laminin- and collagen-comprising basement membrane [11]. VM sanctions tumors to shape matrix-embedded vasculogenic structures, carrying plasma and blood cells to satisfy the metabolic exigencies of swiftly thriving tumors [12]. VM ensues in many intrusive tumors, such as melanomas [13] and breast cancers [14]. Patients with marked VM tumors display a poorer diagnosis [15,16]; VM also positively connects with tumor staging [17]. Presumably, VM plays a causal role in tumor progression, stimulating the invasive growth and metastasis of tumor cells clinically.

In this study, we focused on the role of DPY19L3 for VM and cell proliferation. Knockout (KO) of *DPY19L3* gene resulted in suppression of both VM and proliferation in HT1080 human fibrosarcoma cells. Re-expression of wild-type DPY19L3 restored VM formation and cell growth, whereas expression of DPY19L3-isoform2, an inactive isoform, failed to do so. Moreover, knockdown (KD) of *DPY19L3* decreased VM capability in MDA-MB-231 human breast cancer cells, suggesting that DPY19L3-mediated *C*-mannosylated proteins regulate these phenomena and that DPY19L3 is a new molecular target for cancer therapy.

## Materials and Methods

### Cell culture

Human fibrosarcoma HT1080 (Japanese Collection of Research Bioresources Cell Bank, Osaka, Japan) and human embryonic kidney (HEK) 293T cells were cultured in Dulbecco’s modified Eagle’s medium (DMEM; Nissui Pharmaceutical Co., Ltd., Tokyo, Japan) that was enriched with 7% (v/v) fetal bovine serum (FBS), 100 U/mL penicillin G, 100 mg/L kanamycin, 600 mg/L L-glutamine, and 2.25 g/L NaHCO_3_ at 37°C in a humidified incubator with 5% CO_2_. Human breast cancer MDA-MB-231 cells were cultured in DMEM that was enriched with 15% (v/v) FBS, 100 U/mL penicillin G, 100 mg/L kanamycin, 600 mg/L L-glutamine, and 2.25 g/L NaHCO_3_ at 37°C in a humidified incubator with 5% CO_2_.

### Establishment of DPY19L3-knockout cell lines using the CRISPR/Cas9 system

DPY19L3-knockout (KO) HT1080 cell line was formed with the CRISPR/Cas9 system using formerly reported methods [18,19]. The oligos that were used to create single-guide RNA (sgRNA) were inserted into the BbsI site of the pSpCas9n(BB)-2A-Puro (PX459) V2.0 vector, which was placed into Addgene by Dr. Feng Zhang (#62987, Addgene, Cambridge, MA, USA). This plasmid was altered to exhibit the Cas9 nickase (D10A mutant), which generates a single-strand break in DNA. Thus, we used two close pairs of sgRNAs, and target sequences were designed in exon 6 of human *DPY19L3*. The primers that were used to clone the guide sequences were as follows: 5′-CACCGCTGGCTACTCAGTGGTACA-3′ (forward 1) and 5′-AAACTGTACCACTGAGTAGCCAGC-3′ (reverse 1); 5′-CACCGAGCTGTGACATAGATCGCC-3′ (forward 2) and 5′-AAACGGCGATCTATGTCACAGCTC-3′ (reverse 2). Each pair of primers was annealed and then inserted into the plasmid. The two plasmids were co-transfected into the HT1080 cells, followed by selection with 2 μg/mL puromycin dihydrochloride (Merck KGaA, Darmstadt, Germany). After selection, clonal cell lines were isolated by limiting dilution methods. KO of DPY19L3 in the clone was confirmed by sequence analysis.

### MTT assay

MTT assay was carried out to measure cell proliferation rates with thiazolyl blue tetrazolium bromide (Merck KGaA). After seeding cells in a 96-well plate at 2.0 × 10^3^ cells/well, the cells were cultured at 37°C with 5% CO_2_ for 24 or 48 h. After incubation, 20 mL 0.5 mg/mL MTT was added, and the mixtures were incubated for 4 h at 37°C. After removing the culture supernatant, 100 µL of DMSO was added to each well, and the solution was mixed well with a shaker. The absorbance was calculated at 570 nm using a Multiskan™ FC Microplate Photometer microplate reader (Thermo Fisher Scientific, Inc., Waltham, MA, USA) [20].

### VM-like network formation assay

HT1080 and MDA-MB-231 cells, suspended in culture medium, were seeded at 3.2 × 10^3^ and 2.0 × 10^4^ cells/well, respectively, in a 96-well plate that was precoated with 40 µL/well Matrigel (Corning Inc., Somerville, MA, USA). The seeded cells were cultured at 37°C and photographed under a phase-contrast microscope (Leica DMi1; Leica Microsystems GmbH, Wetzlar, Germany) [21].

### Semiquantitative RT-PCR

Total RNAs were extracted with buffer A (38% (w/v) phenol, 0.8 M guanidine thiocyanate, 0.4 M ammonium thiocyanate, 0.1 M sodium acetate trihydrate, and 5% (v/v) glycerol) and reverse-transcribed using an RT-PCR kit (Thermo Fisher Scientific, Inc.). The resulting cDNAs were used by semiquantitative PCR to evaluate DPY19L3 expression using the following primers: 5′-GGCACAGTTGACCTGAAACC-3′ (forward) and 5′-CTTCCTCTGGTGCCCTCTTG-3′ (reverse). Human *β-actin* was employed as an internal control and amplified with the following primers: 5′-CTTCGAGCACGAGATGGCCA-3′ (forward) and 5′-CCAGACAGCACTGTGTTGGC-3′ (reverse) [22].

### Establishment of DPY19L3-rescued cell lines

Human *DPY19L3* (wt) and *DPY19L3* (isoform2) genes were cloned from pCI-neo-*DPY19L3*-Gluc and pCI-neo-*DPY19L3* (isoform2)-Gluc [23], respectively. To fend off Cas9 identification and deletion of exogenously introduced *DPY19L3* gene, we engendered a Cas9-resistant *DPY19L3* gene by codon optimization without any amino acid swapping. The sequences of the primers, used to amplify the Cas9-resistant *DPY19L3* gene, are as follows:

Forward: 5′-CCTCTACATAACCTCTTGGTTGTTGTCCGGCACCTGGCTGTCAGGACTGTTGGC-3′ and reverse: 5′-GCGGTCACGTAAATGGCTTGCAGCCCAAATAAGGTGTAAATATAAAAATAAACTG-3′. The Cas9-resistant *DPY19L3* gene was subcloned into the XhoI/NotI restriction sites of CSII-CMV-MCS-IRES2-Bsd plasmid (RIKEN BioResource Center, Tsukuba, Japan). For control, Gluc gene from pGluc basic (New England Biolabs, Inc., Ipswich, MA, USA) was subcloned into the EcoRI/NotI restriction sites of CSII-CMV-MCS-IRES2-Bsd plasmid. These plasmids were transfected with Lentivirus High Titer Packaging Mix (Takara Bio Inc., Shiga, Japan) into HEK293T cells for lentivirus production. After 6 h, cells were washed, and fresh media was added. After a supplemental 48 h of culture, the conditioned media containing lentivirus was collected, and *DPY19L3*-KO HT1080 cells were infected with the lentivirus media for 24 h. Infected cells were sorted out with 15 μg/mL blasticidin S (FUJIFILM Wako Pure Chemical Corporation, Osaka, Japan) for 1 week. Reintroduction of *DPY19L3* was validated by western blot.

### Preparation of shRNA expression vector and knockdown of target gene

The shRNA expression vector was constructed using the pLKO.1-TRC cloning vector (Addgene) and the designed shRNA primers within human *DPY19L3*: forward (exon 12) 5′-GGCACAGTTGACCTGAAACC-3′ and reverse (exon 17) 5′-CTTCCTCTGGTGCCCTCTTG-3′. The oligonucleotides were annealed and then inserted into the EcoRI/AgeI restriction site of the pLKO.1-TRC cloning vector. These plasmids were transfected with Lentivirus High Titer Packaging Mix (Takara Bio Inc.) into HEK293T cells for lentivirus manufacturing. After 6 h, cells were washed, and fresh media was added. After a supplemental 48 h of culture, the conditioned media containing lentivirus was collected, and MDA-MB-231 cells were infected with the lentivirus media for 24 h. Infected cells were sorted out with 15 μg/mL blasticidin S (FUJIFILM Wako Pure Chemical Corporation, Richmond, VA, USA) for 1 week.

### Western blot

Cells were lysed in lysis buffer [50 mM Tris–HCl (pH 7.5), 150 mM NaCl, 0.1% (w/v) SDS, 1% (v/v) Triton X-100, 1% (w/v) sodium deoxycholate, and 1 mM phenylmethylsulfonyl fluoride] at 4°C with sonication. To remove pellets, centrifugation at 13,000 rpm was performed. The amount of protein in each lysate was measured by Coomassie Brilliant Blue G-250 staining (Bio-Rad Laboratories, Inc., Hercules, CA, USA). Then, 6 × loading buffer [350 mM Tris–HCl (pH 6.8), 30% (v/v) glycerol, 0.012% (w/v) bromophenol blue, 6% (w/v) SDS, and 30% (v/v) 2-mercaptoethanol] was added. Equal amounts of proteins were loaded onto SDS-polyacrylamide gels, transferred to a PVDF membrane, and blocked with skim milk. The membranes were immunoblotted with anti-Gluc (used to detect DPY19L3), anti-Akt, anti-P-Akt (rabbit polyclonal, #8023S, New England Biolabs, Inc., Ipswich, MA, USA) and α-tubulin (mouse monoclonal, #T5168, Merck KGaA). Signals were discerned with ECL using Western Lightning Plus-ECL (PerkinElmer, Inc., Waltham, MA, USA) [24].

### Proliferation assay

Control, *DPY19L3* wt- and *DPY19L3* isoform2-overexpressing HT1080 cells were seeded at 1.0 × 10^3^ cells/well in 12-well plates and cultured for 3 h. Technically, the time after 3 h was considered 0 h. The 3 h incubation was only used to give more time for the cells to proliferate after seeding. After 3 h, cells were recovered by trypsinization and counted using a hemacytometer. The same process was done when the cells were counted at 72 h.

## Results

Knockout of *DPY19L3* inhibits VM formation and proliferation in HT1080 cells.

**Figure 1:**
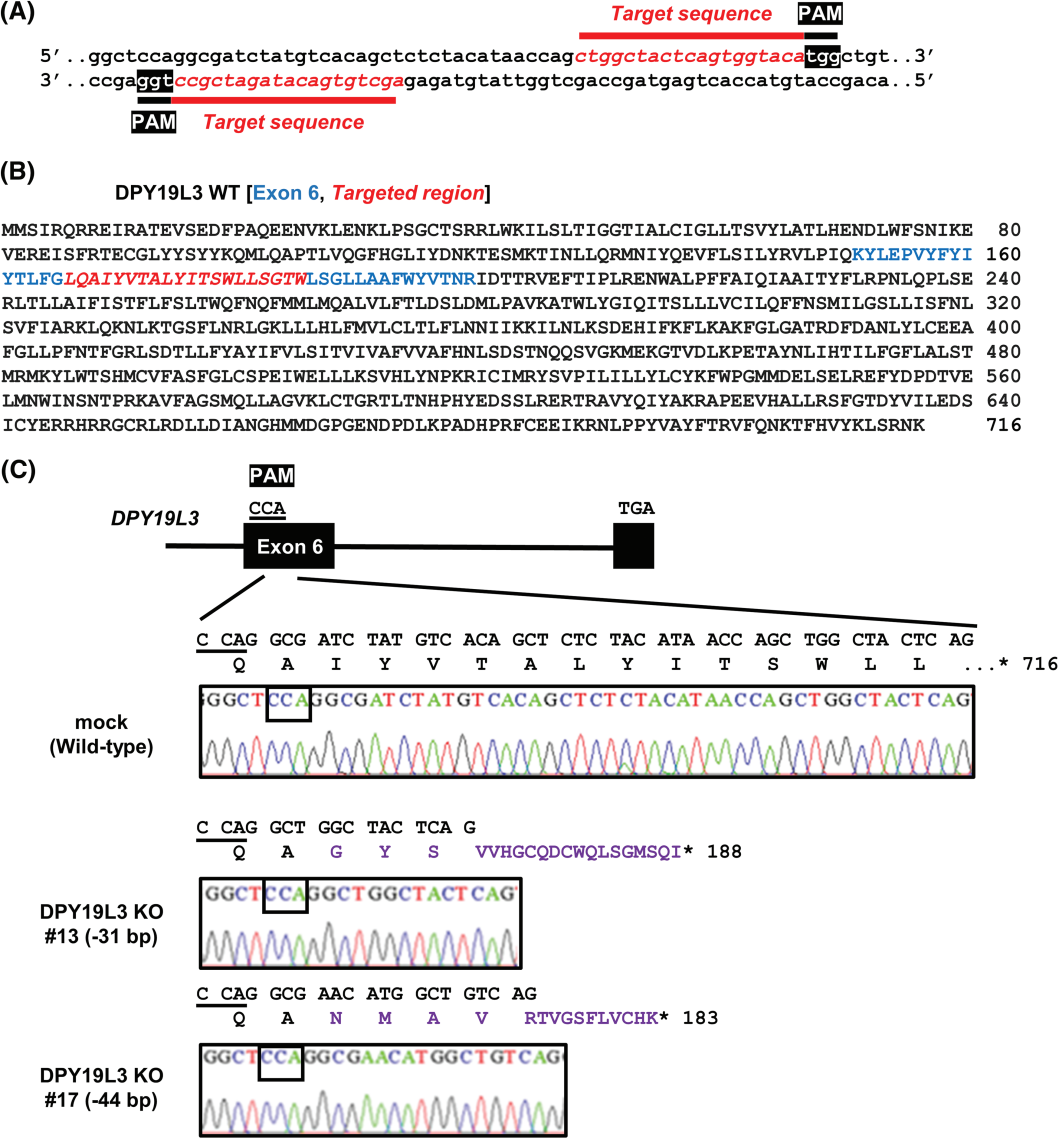
Establishment of HT1080-*DPY19L3* KO cells. (A) The target sequence of human *DPY19L3* gene. The protospacer-adjacent motif (PAM, white) and target (italic red) sequences are shown. (B) The amino acid sequence of human DPY19L3. Human DPY19L3 comprises 716 amino acids. The exon 6 (blue) and target region (Italic red) are indicated. (C) The genomic sequence of *DPY19L3* from HT1080 cells. CRISPR–Cas9-induced mutation detected by amplicon sequencing in *DPY19L3*. The exons (boxes) and intron (line) indicate the schematic arrangement of the *DPY19L3* gene. The genomic sequence of *DPY19L3* from HT1080 (mock) was shown as wild-type (WT). The 31- and 44-bp deletions were observed in *DPY19L3* KO #13 and #17 cells, respectively. *codon stop.

To examine the function of DPY19L3 in VM genesis, we sought to establish HT1080 cell lines with deleted human *DPY19L3* gene using CRISPR/Cas9 system, because we have reported that *DPY19L3* is expressed in HT1080 cells [8] and HT1080 cells have enough VM formation capability [21]. Guide RNA sequences were designed at 2 close positions in exon 6 of DPY19L3 (Figs. 1A and 1B). We obtained two independent clones (#13 and #17), analyzed genetic alterations, and detected frameshift mutations generating immature codon stop in the cell lines, compared with HT1080 cells transfected with empty vector (mock) (Fig. 1C). Because there is no suitable antibody to detect endogenous DPY19L3, we could not confirm the KO of *DPY19L3* by western blot. However, we could find frameshift mutations generating immature stop codon within *DPY19L3* gene; these clonal cell lines were designated as *DPY19L3*-KO cells.

**Figure 2:**
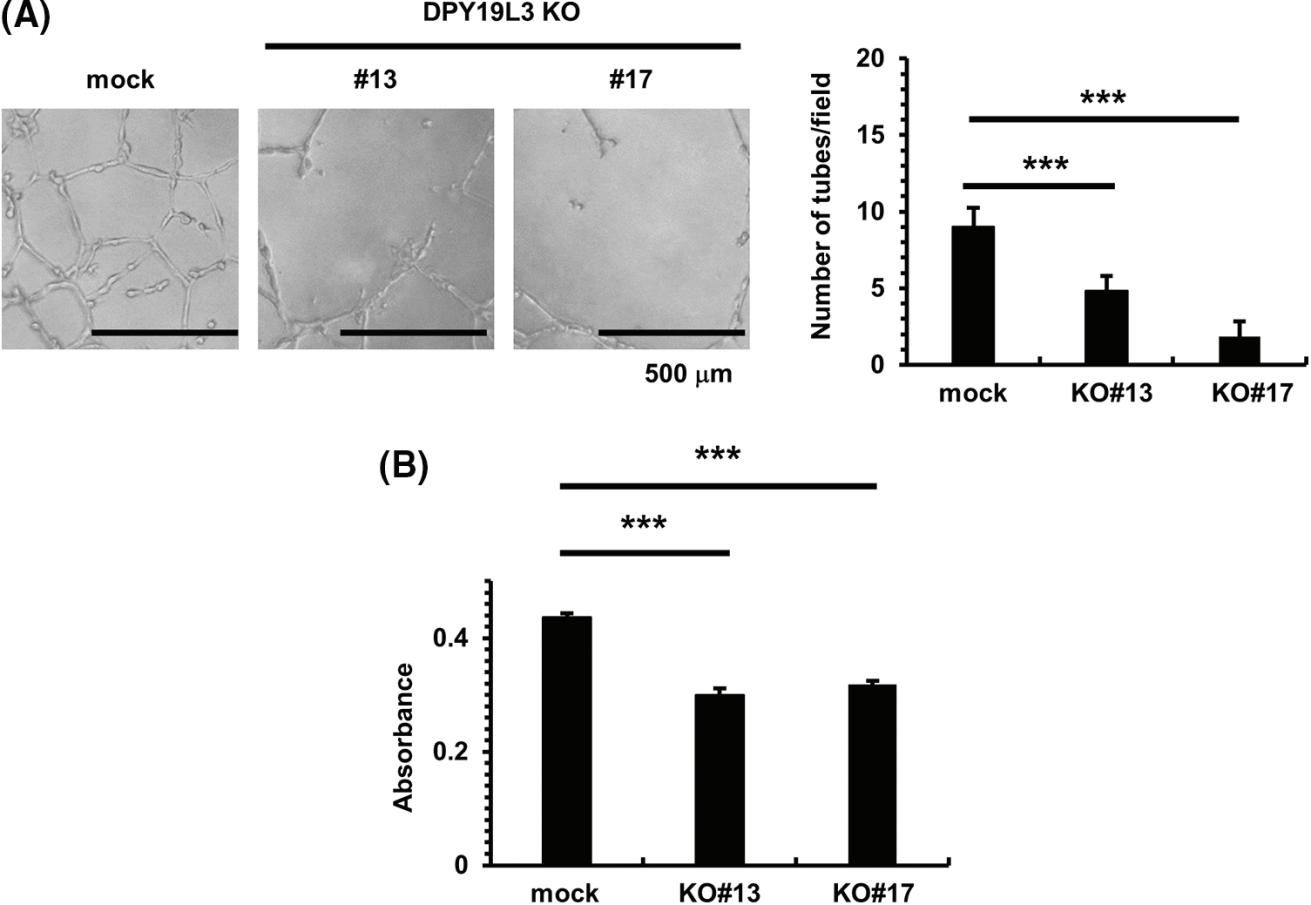
DPY19L3 KO suppressed VM-like network formation and proliferation. (A) VM was abolished by KO of *DPY19L3*. VM formation assay was performed, and the tube numbers were photographed (left) and counted (right) 24 h after seeding. Empty vector (mock) was used as a control. Scale bar, 500 µm. (B) Effect of DPY19L3 KO on the cell proliferation of HT1080 cells. Cells were seeded on 96 well plates at 2.0 × 10^3^ cells/well, and MTT assay was performed at 24 h. Data shown are means ± SD (n = 5). ***p-values < .001.

To investigate whether DPY19L3 is an important factor for VM formation, we seeded *DPY19L3*-KO HT1080 cells on Matrigel which resembles the laminin/collagen IV-rich basement membrane extracellular environment found in many tissues. Matrigel is used because cancer cells form VM only on it. After 24 h, HT1080 mock cells formed VM, as expected; however, the network formation was significantly inhibited in both *DPY19L3* KO cell lines (Fig. 2A). We checked the relation between DPY19L3 and cell proliferation. As shown in Fig. 2B, *DPY19L3*-KO cell lines proliferated slower than HT1080 mock cells. Thus, these results suggested that DPY19L3 contributes to both VM formation and proliferation.

DPY19L3 regulates VM formation and proliferation via its C-mannosyltransferase activity.

**Figure 3:**
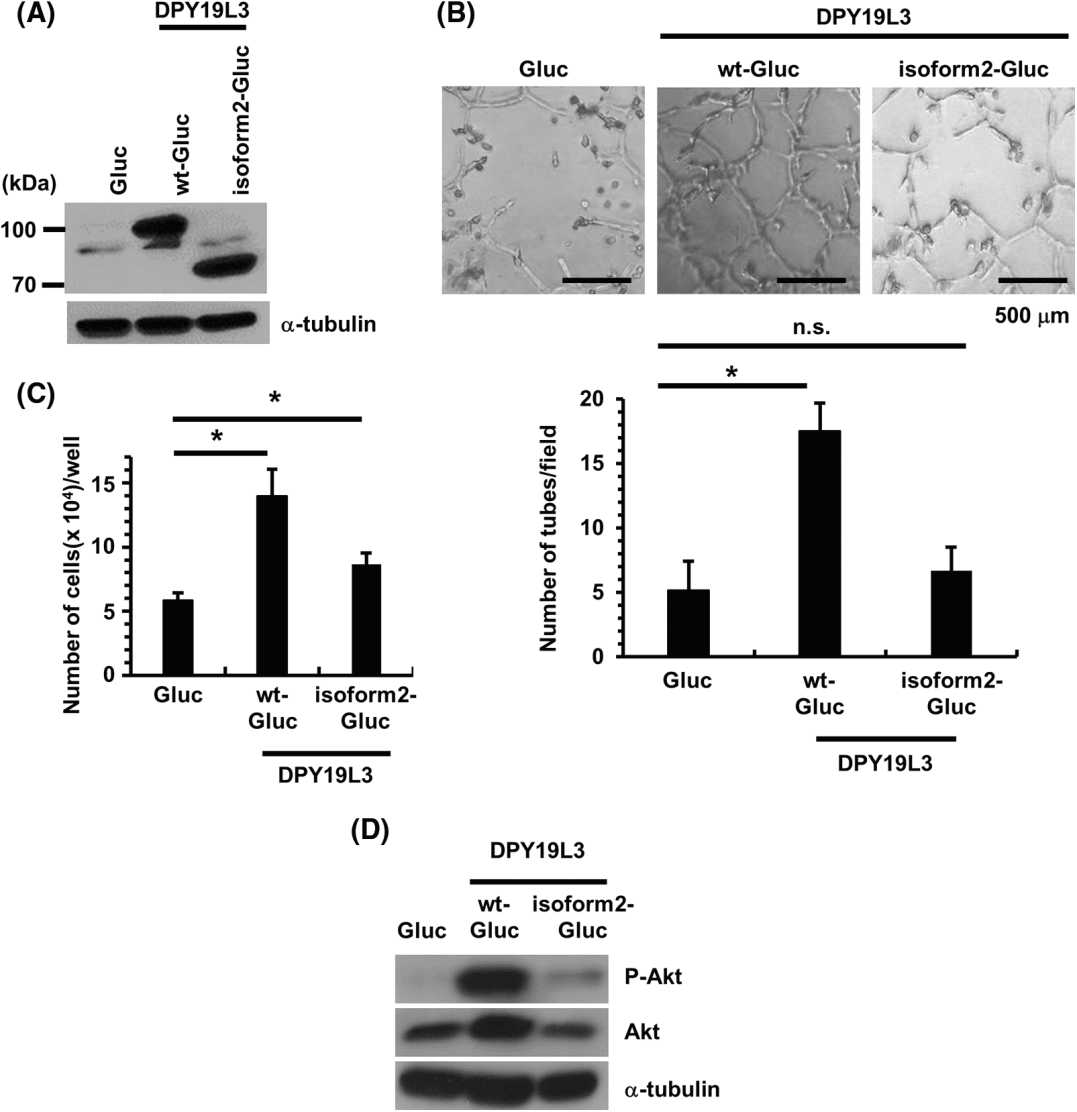
Re-expression of *DPY19L3* wt but not isoform2 recovered the VM-like network genesis ability of HT1080-*DPY19L3* KO cells. (A) Re-expression of Gluc (control), *DPY19L3*-wt-Gluc, or *DPY19L3*-isoform2-Gluc in HT1080-*DPY19L3* KO#13 cells. The cells were lysed and immunoblotted with DPY19L3 antibody. (B) Expression of *DPY19L3* in Gluc, DPY19L3-wt-Gluc, and DPY19L3-isoform2-Gluc HT1080 cells. The result of re-expression of DPY19L3 on VM formation. Cells were seeded at 3.2 × 10^3^ cells/well on Matrigel-coated 96-well plates, and photographs were taken 72 h after seeding (upper). The number of tubes was enumerated in 6 arbitrarily selected, independent fields (lower). Data shown are means ± SD. (C) The effect of re-expression of DPY19L3 on cell proliferation. Control, *DPY19L3* wt-, and DPY19L3 isoform2-overexpressing HT1080 cells were seeded at 1.0 × 10^3^ cells/well in 12-well plates; the cells were counted at 72 h. n.s.; not significant. Data shown are means ± SD (n = 5). (D) The expression of Akt in DPY19L3 rescue cells. DPY19L3 overexpressed cells were lysed and immunoblotted with Akt and phosphorylated Akt antibodies. *p-values < .05.

According to previous studies, isoform2, a splicing variant of DPY19L3, has the same amino acid sequence and topology as the wild-type except that most of the C-terminal vesicle endoplasmic reticulum domain is deleted. Isoform2 is not known to exhibit any C-mannosyltransferase activity [23]. Therefore, to examine the effect of the enzymatic activity of DPY19L3 on VM formation, we re-expressed wild-type and isoform2 with the Gluc tag attached to the C-terminus for western-blot detection (Fig. 3A) and evaluated VM. As a result, the VM formation was restored by expression of wild-type DPY19L3-expressing cells but failed to recover in isoform2-expressing cells (Fig. 3B). We also investigated the effect of *C*-mannosyltransferase enzymatic activity on cells’ proliferation. Wild-type DPY19L3-expressing cells multiplied more than DPY19L3 mock and isoform2 cells (Fig. 3C). From the results above, it is suggested that the *C*-mannosyltransferase activity of DPY19L3-mediated *C*-mannosylated proteins may contribute to the formation of VM and proliferation of HT1080 cells. When comparing the expression of Akt and phosphorylated Akt in DPY19L3 overexpressed cells, it was ascertained that this expression is higher in WT DPY19L3 overexpressed cells as compared to Gluc and isoform2 overexpressed cells (Fig. 3D).

### DPY19L3 also contributes to VM-like network genesis in MDA-MB-231 cells

**Figure 4:**
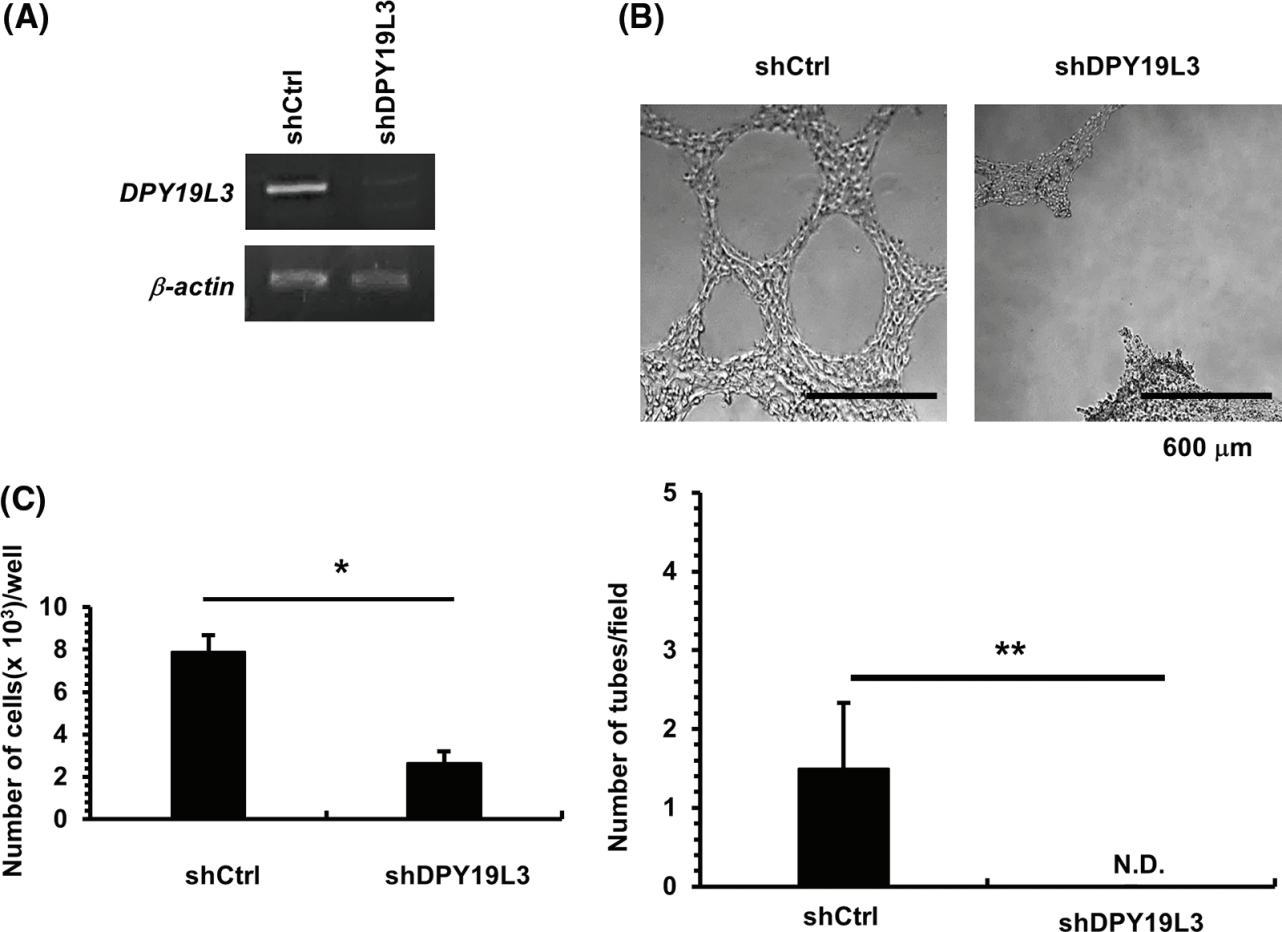
The effect of DPY19L3 knockdown on VM genesis in MDA-MB-231 cells. (A) Expression of *DPY19L3* in shRNA-transfected MDA-MB-231 cells. (B) Effect of *DPY19L3* knockdown on VM formation in MDA-MB-231 cells. Cells were seeded at 2.0 × 10^4^ cells/well on Matrigel-coated 96-well plates, and photographs were taken at 24 h after seeding. The number of tubes was enumerated in 6 arbitrarily selected, independent fields. N.D.; not detected. (C) The effect of DPY19L3 knockdown on cell proliferation. Control and KD cells were seeded at 1.0 × 10^3^ cells/well in 12-well plates; the cells were counted at 24 h. Data shown are the means ± SD (n = 5). *p-values < .05, **p-values < .01.

As previously shown, DPY19L3 is required for VM formation, but since sarcoma derived from HT1080 cells is a cancer with a low developing probability, the range of the findings obtained in this study that can be applied is bounded. To broaden our study, we centered our attention on the function of DPY19L3 in breast cancer. KD of DPY19L3 was performed in breast cancer-derived MDA-MB-231 cells by gene transfer of shRNA targeting DPY19L3, and we confirmed it (Fig. 4A). As shown in Fig. 4B, VM capability of MDA-MB-231 cells was dramatically decreased in DPY19L3-KD cells compared with cells transfected with GFP-targeted shRNA (shCtrl). In addition, DPY19L3-KD cells proliferated less than control cells (Fig. 4C).

## Discussion

Metastasis, a crucial cancer step, is the expansion of cancer cells to tissues and organs beyond where the tumor started, and the genesis of new tumors is the single event which outcome is the death of most patients with cancer [25]. Thus, metastasis is the most life-threatening event in patients with cancer and is a target for cancer therapeutics. The loss of cell-cell adhesion capacity empowers malignant tumor cells to disconnect from the primary tumor mass, and modifications in cell-matrix interaction sanction the cells to conquer the adjacent stroma. This involves the emission of substances to break down the basement membrane, the extracellular matrix and the expression/suppression of proteins incriminated in the control of motility and migration. The tumor must also carry out angiogenesis, without which the tumor would fail to develop [26]. Angiogenesis is the process by which tumors recruit new blood vessels from the existing circulation or from surrounding stromal cells. On the other hand, VM is a recently discovered process by which tumors build up a highly patterned microcirculation that is independent of angiogenesis: in aggressive primary and metastatic melanomas, the tumor cells give rise to acellular microcirculatory channels [27]. Since therapeutics against angiogenesis are not sufficient for a cure, VM has been an attractive target for cancer therapeutics. However, the detailed mechanism underlying VM is not fully understood.

*C*-mannosylation is a structurally diverse and convoluted posttranslational modification. In addition to the structural modification of extracellular and cell membrane proteins, intracellular proteins can also be glycosylated, with functional implications. This posttranslational modification has regulatory functions akin to other modifications, modulating protein conformation, steadiness, and reversible multimeric protein assembly. Abnormal glycosylation is an essential part of all recognized cancer hallmark traits [28]. In this study, we wanted to unravel the relationship between *C*-mannosylation and VM, as it has not yet been reported.

In this report, we found that DPY19L3 knockout markedly suppressed VM formation in HT1080 cells (Fig. 2). Furthermore, re-expression experiments showed that VM formation was dependent on the enzymatic activity of DPY19L3 (Fig. 3). Therefore, it is suggested that the *C*-mannosylated proteins catalyzed by DPY19L3 positively regulate VM formation. We stipulate that the modified proteins assist cancer cells in their VM formation. Moreover, the proliferation of HT1080 cells was also shown to be dependent on the enzymatic activity of DPY19L3. At present, we do not know whether the *C*-mannosylated proteins involved in VM and proliferation are the same; hence, it is needed to elucidate the responsible substrate proteins in the future. Furthermore, KD of DPY19L3 also suppressed VM formation in MDA-MB-231 human breast cancer cells (Fig. 4). Using GEPIA, we checked the expression levels of DPY19L3 in cancer and normal cells; however, we could not notice any discernible relationship. Hence, we speculate that the substrate(s) and not DPY19L3 itself might be important for VM formation and cell proliferation. Akt is a serine/threonine kinase that has a key role in paramount cellular functions including cell size, cell cycle, transcription, and neovascularization. Akt nurtures cell survival by arbitrating the cellular growth factors and blocking apoptosis through the inactivation of pro-apoptotic proteins [29]. Phosphorylated Akt bestows the phosphorylation of different proteins located either in the plasma membrane, in the nucleus, or in the cytosol, promoting cell growth and survival, among other cellular effects. Downstream targets of Akt phosphorylation heighten tumor motility and invasion [30,31]. Since the expression of Akt and phosphorylated Akt is higher in WT DPY19L3 overexpressed cells, we stipulate the importance of the Akt pathway for these cells. These findings suggest that DPY19L3 is a novel promising molecular target for cancer therapy. On the other hand, we believe that different components of the Akt pathway should be analyzed to decipher any correlation with DPY19L3-moderated *C*-mannosylation. In addition, it has been reported that DPY19L1 also has enzymatic activity [9]; so, it seems necessary to investigate the involvement of DPY19L1 in VM formation. In summary, DPY19L3-mediated *C*-mannosylation is a novel auspicious cancer molecular target, as our results suggest. The emphasis should be on the inhibition of DPY19L3, and the development of small-molecule inhibitors of DPY19L3 is desired, as our results strongly suggest such inhibition.

## Data Availability

Data are available upon reasonable request.
